# Effect of Anticoagulant Administration on the Mortality of Hospitalized Patients With COVID-19: An Updated Systematic Review and Meta-Analysis

**DOI:** 10.3389/fmed.2021.698935

**Published:** 2021-08-04

**Authors:** Luojia Jiang, Yupei Li, Heyue Du, Zheng Qin, Baihai Su

**Affiliations:** ^1^Department of Nephrology, West China Hospital, Sichuan University, Chengdu, China; ^2^Department of Nephrology, Jiujiang No. 1 People's Hospital, Jiujiang, China; ^3^Disaster Medicine Center, Institute for Disaster Management and Reconstruction, Sichuan University, Chengdu, China; ^4^The First People's Hospital of Shuangliu District, Chengdu, China; ^5^Med-X Center for Materials, Sichuan University, Chengdu, China

**Keywords:** anticoagulation, coronavirus disease 2019, heparin, mortality, bleeding

## Abstract

**Background:** Anticoagulation is generally used in hospitalized patients with coronavirus disease 2019 (COVID-19) as thromboprophylaxis. However, results from different studies comparing the effect of anticoagulation on the mortality of COVID-19 patients with non-anticoagulation are inconclusive.

**Methods:** Our systematic review included observational trials if they studied anticoagulant therapy in hospitalized patients with COVID-19 for mortality or bleeding events. Dichotomous variables from individual studies were pooled by risk ratio (RR) and their 95% confidence interval (95% CI) using the random-effects model. Grading of Recommendations Assessment, Development and Evaluation was used to assess the quality of evidence.

**Results:** A total of 11 observational studies enrolling 20,748 hospitalized COVID-19 patients overall were included. A pooled meta-analysis of these studies showed that anticoagulation therapy, compared with non-anticoagulation therapy, was associated with lower mortality risk (RR 0.70, 95% CI 0.52–0.93, *p* = 0.01). The evidence of benefit was stronger among critically ill COVID-19 patients in the intensive care units (RR 0.59, 95% CI 0.43–0.83, *p* = 0.002). Additionally, severe bleeding events were not associated with the administration of anticoagulants (RR 0.93, 95% CI 0.71–1.23, *p* = 0.63).

**Conclusion:** Among patients with COVID-19 admitted to hospital, the administration of anticoagulants was associated with a decreased mortality without increasing the incidence of bleeding events.

## Introduction

Coronavirus disease 2019 (COVID-19), provoked by severe acute respiratory syndrome coronavirus-2 (SARS-CoV-2) infection, was first reported in December 2019 and is the most serious worldwide public health crisis (1, 2). Venous thromboembolism (VTE) is the most commonly reported thrombotic complication, with a high incidence rate of 27.9% among critically ill COVID-19 patients admitted to intensive care units (ICUs)

(3, 4). The incidence of pulmonary embolism in patients with COVID-19 who underwent pulmonary CT angiography was reported to be between 22 and 30% (2, 5). Accordingly, even though the risk–benefit ratio of anticoagulation has not been established clearly, some guidelines have recommended prophylactic dose anticoagulation for COVID-19 patients who do not have a contraindication to this treatment to reduce the risk of VTE (6–8).

Some retrospective observational studies showed that anticoagulant administration was associated with reduced mortality (9, 10), but others did not confirm these findings and, rather, suggested an elevated risk of bleeding (11–13). Limited evidence exists to guide the prophylactic antithrombotic regimen in COVID-19 patients due to a lack of randomized clinical trials. Up to date, the cumulative number of inpatients with COVID-19 in cohort studies exploring the effect of anticoagulation therapy on the mortality has exceeded 20,000. In this study, we set out to perform an updated meta-analysis of current evidence to further clarify whether hospitalized patients with COVID-19 benefit from anticoagulation therapy (including both therapeutic– and prophylactic–dose anticoagulation therapy).

## Methods

This meta-analysis followed the Preferred Reporting Items for Systematic Reviews and Meta-Analyses (PRISMA) Statement (14), with the PRISMA checklist provided in Supplementary Table 1, and was registered in the Open PROSPERO Framework (CRD42021229707). All steps were performed independently by two investigators (JLJ and LYP). Any discrepancies were discussed with the corresponding author (SBH).

### Study Selection Criteria

Two reviewers independently screened all relevant articles published on MEDLINE, the Cochrane Library, EMBASE, and the Web of Science databases from inception to March 28, 2021. The search terms used were “COVID-19,” “2019 novel coronavirus infection,” “coronavirus disease-2019,” “2019-nCoV disease,” “2019 novel coronavirus disease,” “severe acute respiratory syndrome coronavirus 2,” “Wuhan coronavirus,” “Wuhan seafood market pneumonia virus,” “SARS-CoV-2,” “SARS2,” “anticoagulant,” “anticoagulation,” “heparin,” “unfractionated heparin,” “UFH,” “fondaparinux,” “enoxaparin,” “low-molecular-weight heparin,” “heparin, low molecular weight,” “LMWH,” “thromboprophylaxis,” “antithrombotic,” and “anti-thrombosis” (see Supplementary Table 2 for the detailed search strategy). The titles and abstracts of the resulting articles were examined to exclude irrelevant studies. The full texts of the remaining articles were read to determine if these articles meet our eligibility criteria. Randomized controlled trials (RCTs) or observational studies that compared the effect of anticoagulation vs. non-anticoagulation on the mortality and/or bleeding events of hospitalized COVID-19 patients were included. The exclusion criteria were as follows: (1) absence of original data; (2) enrollment of non-hospitalized COVID-19 patients; (3) case series/report; (4) absence of a comparator group; and (5) non-human studies.

### Data Extraction and Quality Assessment

This meta-analysis mainly investigated the impact of anticoagulation on the in-hospital mortality and bleeding risk of inpatients with COVID-19. The Newcastle–Ottawa Scale was used to assess the methodological quality of observational studies (case–control or cohort studies), which has eight criteria and yields scores ranging from 0 to 9. Studies with scores ≥7 were regarded as high quality (15). The Grading of Recommendations Assessment, Development and Evaluation (GRADE) framework was applied for rating the quality of evidence for each outcome in the pooled analysis, and the quality of evidence was rated as high, moderate, low, and very low quality (16). The credibility of results from subgroup analyses was assessed by specific criteria (17, 18).

### Outcomes

The primary outcome was all-cause in-hospital mortality during hospitalization for COVID-19. The secondary outcome was the incidence of bleeding events during hospitalization for COVID-19. Definition of bleeding events was according to definitions in the individual studies, mainly including (1) an active source of bleeding; (2) suspected bleeding without confirmation of an active bleeding source; (3) confirmatory imaging or other evidence (neuroimaging for intracranial bleed); and (4) bleeding necessitating a transfusion of packed red blood cells.

### Statistical Analysis

Dichotomous variables such as mortality or bleeding events were expressed as risk ratio (RR) and 95% confidence interval (CI). Heterogeneity was evaluated using the *I*^2^ statistic and Cochrane Q test to assess the degree of inter-study variation. *I*^2^ values of 0–24.9%, 25–49.9%, 50–74.9%, and 75–100% were considered as having no, mild, moderate, and significant thresholds for statistical heterogeneity, respectively (19, 20). A random-effects model using restricted maximum likelihood (21), which is thought to be better than the conventional DerSimonian–Laird method (22), was performed to pool the data due to potential clinical heterogeneity. Potential publication bias was assessed by inspection of funnel plots when the total number of included studies surpasses 10, with Begg's rank correlation test performed subsequently. Predefined sensitivity analyses were conducted using a random-effects model and a leave-one-out approach. We also conducted two exploratory sensitivity analyses by excluding the clinical trials performed in the USA and by excluding those performed outside the USA in the meta-analysis of mortality, respectively. All statistical analyses were performed using Stata version 12.0 and Review Manager version 5.3.

## Results

### Eligible Studies

**Figure 1:**
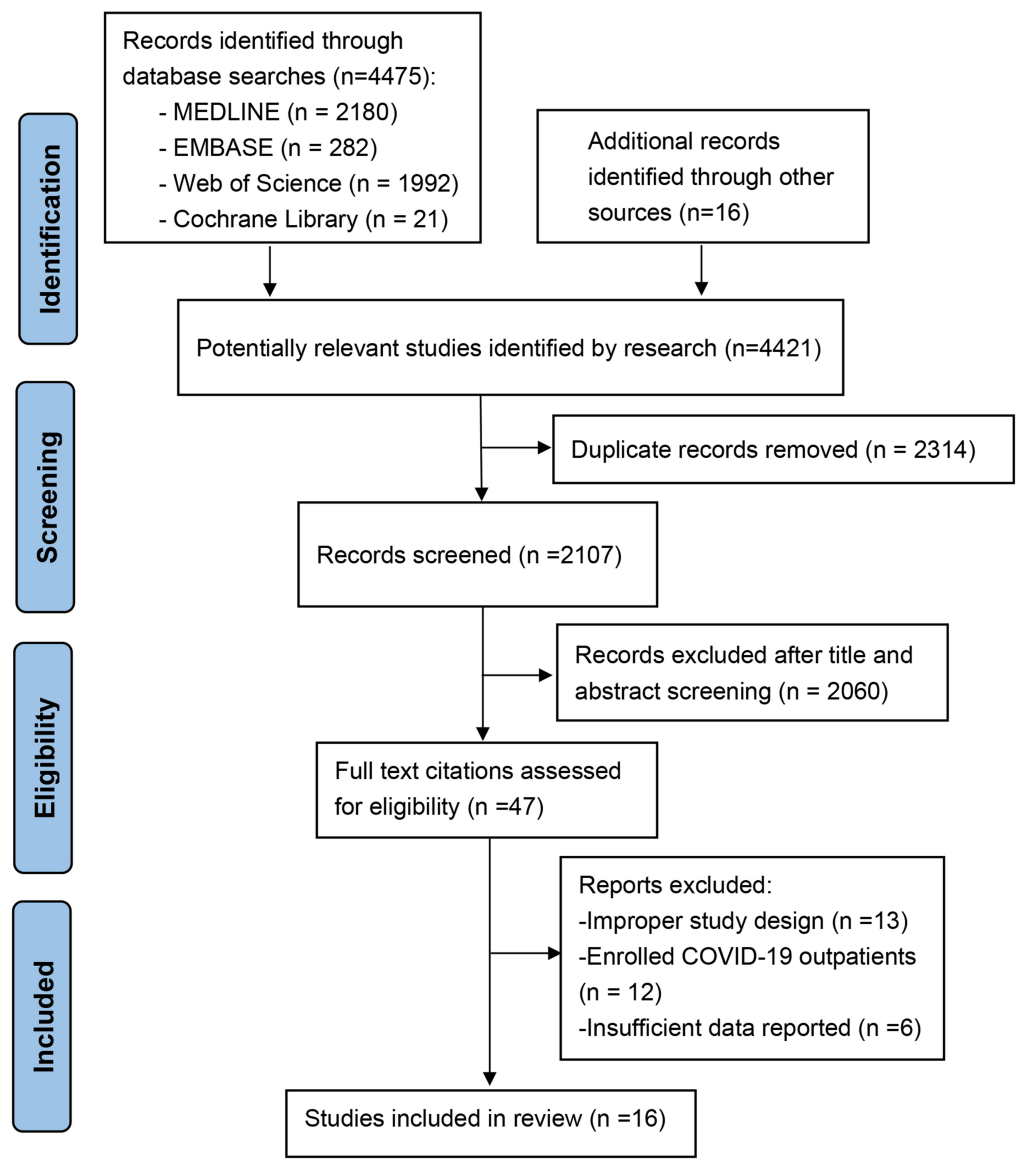
Flowchart of selection of studies.

The study selection process is presented in Figure 1. The literature search yielded 4,421 potentially relevant records. We retained 2,107 relevant studies after removing duplicate studies. After we evaluated the abstract and title of each record, 2,060 studies were excluded because they did not meet the inclusion criteria or they met the exclusion criteria. Of the remaining 47 studies, 36 were excluded after reviewing the full-text manuscript (10 used direct oral anticoagulants as a control group, eight did not compare the mortality of COVID patients receiving anticoagulation with those not receiving anticoagulation directly, 12 enrolled outpatients, and six did not have insufficient outcome data or baseline data for further meta-analysis). A total of 11 observational studies reporting the effect of anticoagulants on mortality (9–11, 23–30) (*n* = 11) and bleeding events (9, 25, 27–29) (*n* = 5) in hospitalized patients with COVID-19 were included in the review.

This meta-analysis is based on a pooled sample of 20,748 hospitalized COVID-19 patients with reported information related to mortality and a pooled sample of 11,302 patients with reported information related to bleeding events. The sample sizes ranged widely across studies from 81 to 4,389. All studies were retrospective in study design. Among the included studies, seven were conducted in the USA (9, 10, 25, 27–30), two studies were conducted in China (11, 23), one study was conducted in Spain (26), and one study was conducted in Italy (24). Three studies were conducted exclusively in the ICU settings (9, 11, 24). The mean age in most studies was over 60 years. The proportion of male subjects ranged from 47 to 69%. Heparin and enoxaparin were the most used anticoagulants in the included studies. Table 1 further summarizes the detailed characteristics of each study, and anticoagulation doses and routes are listed in Supplementary Table 3.

**Table 1:** The detailed characteristics of each study.

**References**	**Country**	**Study design**	**Sample size (*n*)**	**Mean age (years)**	**Male (%)**	**ICU or non-ICU stay**	**Conducting D-dimer stratification**	**Reporting bleeding events**	**Confounder adjustment**
Ayerbe et al. (26)	Spain	Retrospective	2,075	67.5	60.5	**×**	**×**	**×**	**×**
Di Castelnuovo et al. 2021 (24)	Italy	Retrospective	2,574	66.5	62	**√**	**√**	**×**	**√**
Daughety et al. (27)	USA	Retrospective	192	61.5	37	**×**	**×**	**√**	**×**
Reyes Gil et al. (30)	USA	Retrospective	217	63	58	**×**	**×**	**×**	**×**
Hsu et al. (28)	USA	Retrospective	468	65	54.9	**×**	**×**	**√**	**√**
Ionescu et al. (25)	USA	Retrospective	3,480	64.5	48.5	**√**	**×**	**√**	**√**
Nadkarni et al. (29)	USA	Retrospective	4,389	65	56	**×**	**×**	**√**	**√**
Paranjpe et al. (9)	USA	Retrospective	2,773	60	28	**√**	**×**	**√**	**√**
Rentsch et al. (10)	USA	Retrospective	4,297	69	92.5	**×**	**×**	**×**	**√**
Tang et al. (11)	China	Retrospective	449	65	56.7	**√**	**√**	**×**	**×**
Zhang et al. (23)	China	Retrospective	81	61	62	**×**	**×**	**×**	**×**

*D-dimer stratification means that the disease severity of COVID-19 patients was stratified by the level of D-dimer to identify the effect of anticoagulation on in-hospital mortality of patients in different subgroups*.

### Quality of Eligible Studies

The Newcastle–Ottawa Scale score for the included studies ranged from 6 to 9, while 10 studies scored above 7 and were of high quality. A full assessment is shown in Table 2.

**Table 2:** Methodological quality of non-randomized studies assessed by the Newcastle–Ottawa Scale.

**References**	**Selection**				**Comparability**	**Outcome/exposure**			**Overall**
	**Representativeness**	**Selection of non-exposed**	**Ascertainment of exposure**	**Demonstration that outcome not present at start**		**Assessment**	**Follow-up**	**Adequacy**	
Ayerbe et al. (26)	–^a^	^*^ ^b^	^*^	^*^	^*^	^*^	^*^	^*^	7
Di Castelnuovo et al. (24)	^*^	^*^	^*^	–^*^	^*^	^*^	^*^	–	7
Daughety et al. (27)	^*^	^*^	^*^	^*^	^*^	^*^	–	–	6
Reyes Gil et al. (30)	^*^	^*^	^*^	^*^	^*^	^*^	^*^	–	7
Hsu et al. (28)	^*^	^*^	^*^	^*^	^**^	^*^	–	^*^	8
Ionescu et al. (25)	^*^	^*^	^*^	^*^	^**^	^*^	–	^*^	8
Nadkarni et al. (29)	^*^	^*^	^*^	^*^	^**^	^*^	^*^	^*^	9
Paranjpe et al. (9)	^*^	^*^	^*^	^*^	^**^	^*^	^*^	–	8
Rentsch et al. (10)	^*^	^*^	^*^	^*^	^**^	^*^	^*^	–	8
Tang et al. (11)	^*^	^*^	^*^	^*^	^*^	^*^	^*^	–	7
Zhang et al. (23)	^*^	^*^	^*^	^*^	^*^	^*^	^*^	–	7

a *No star awarded;*

b *one star awarded;*

* *represents 1 score;*

** *represents 2 score*.

### Meta-Analysis of Mortality

**Figure 2:**
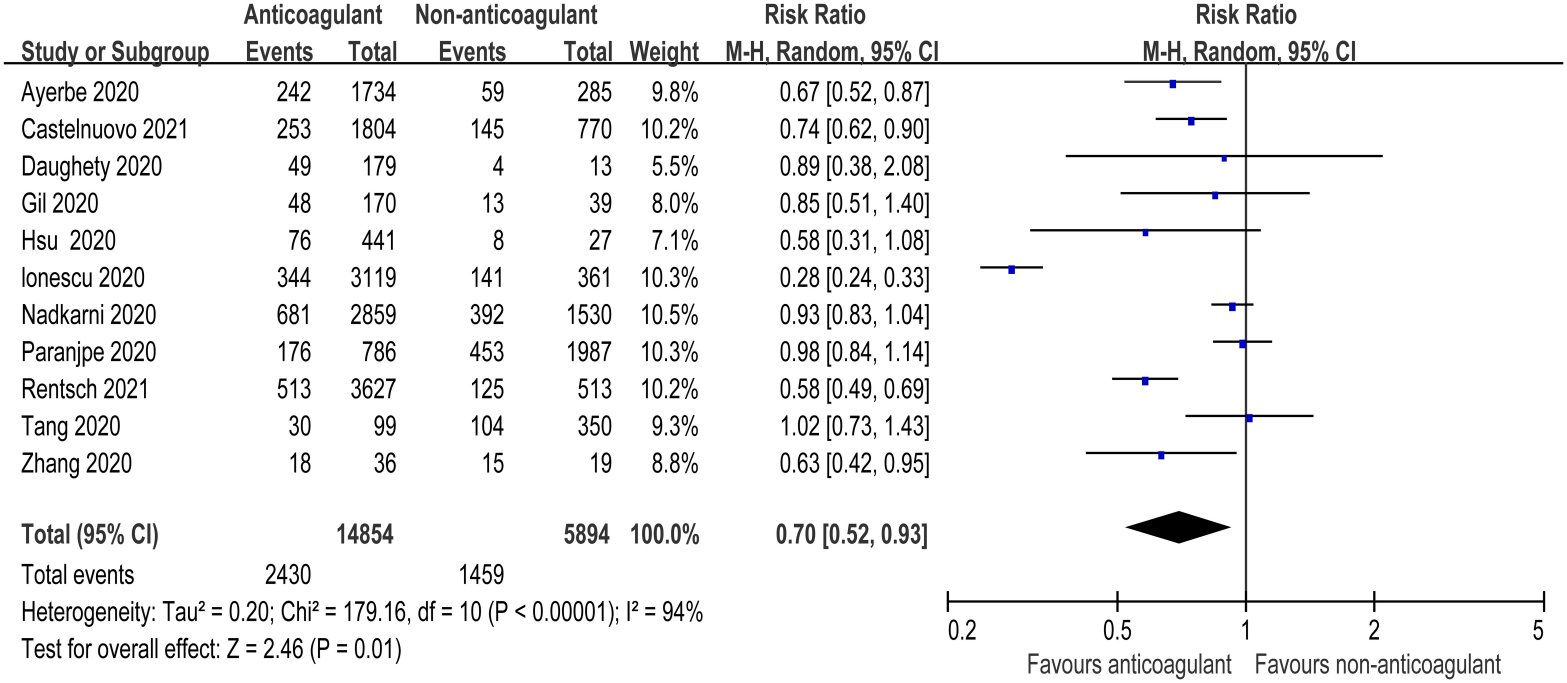
Forest plot of the effect of anticoagulants on risk of mortality in hospitalized COVID-19 patients.

Eleven studies reported the overall mortality of COVID-19 patients receiving prophylactic/therapeutic dose anticoagulation therapy or not receiving anticoagulation, which ranged widely across studies from 8.1 to 27.3% for anticoagulation group and 16.4–29.6% for non-anticoagulation group in mild-to-moderate COVID-19 patients, while it ranged from 29.8 to 40% vs. 62.7 to 78.9% in severe to critical COVID-19 patients, respectively (9–11, 23–30). Of 14,854 hospitalized COVID-19 patients receiving anticoagulant administration, 2,430 patients died during hospitalization, whereas 1,459 of 5,894 patients who did not receive anticoagulant administration died. Figure 2 shows that anticoagulation therapy was significantly associated with reduced in-hospital mortality in inpatients with COVID-19 (RR = 0.70, 95% CI 0.52–0.93, *p* = 0.01, *I*^2^ = 94%). Using the GRADE framework, we rated the quality of evidence in mortality as high (Table 3).

**Table 3:** GRADE quality assessment.

**Certainty assessment**							**No. of patients**		**Effects**		**Certainty**
**No. of participants (studies)**	**Study design**	**Risk of bias**	**Inconsistency**	**Indirectness**	**Imprecision**	**Other considerations**	**Anticoagulation**	**Control**	**Relative (95% CI)**	**Absolute (95% CI)**	
**Mortality in hospitalized patients with COVID-19**											
						Strong association; all plausible residual	14,854 cases 5,894 control				
20,748 (11)	Observational studies	Not serious	Not serious	Not serious	Not serious	confounding would reduce the demonstrated effect	16.4%	24.8%	RR 0.70 (0.52–0.93)	74 fewer per 1,000 (from 119 fewer to 17 fewer)	⊕⊕⊕⊕ High
**Mortality in COVID-19 hospitalized patients admitted to ICU**											
							1,781 cases 891 control				
2,672 (3)	Observational studies	Not serious	Not serious	Not serious	Not serious	Strong association	15.6%	29.4%	RR 0.59 (0.43– 0.83)	121 fewer per 1,000 (from 168 fewer to 50 fewer)	⊕⊕⊕○ Moderate
**Bleeding events in hospitalized patients with COVID-19**											
							7,384 cases 3,918 control			–	
11,302 (5)	Observational studies	Not serious	Not serious	Not serious	Not serious	Strong association	3.1%	2.2%	RR 0.93 (0.71–1.23)	2 fewer per 1,000 (from 6 fewer to 5 more)	⊕⊕⊕○ Moderate

**Figure 3:**
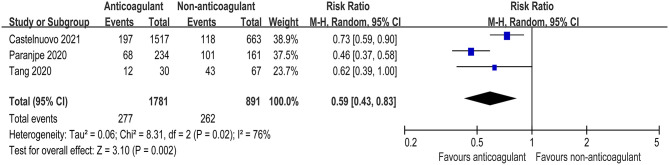
Forest plot of the effect of anticoagulants on risk of mortality in COVID-19 patients admitted to intensive care unit (ICU).

Three studies further reported the mortality in COVID-19 patients admitted to ICU (9, 11, 24). There was also a significant decrease in mortality risk in critically ill COVID patients receiving anticoagulant therapy compared with those not receiving anticoagulant therapy (RR = 0.59, 95% CI 0.43–0.83, *p* = 0.002, *I*^2^ = 76%, Figure 3). The quality of evidence for mortality in hospitalized patients admitted to ICU was rated as moderate because plausible confounding is not considered to reduce or increase demonstrated effect (Table 3).

### Meta-Analysis of Bleeding Events

**Figure 4:**
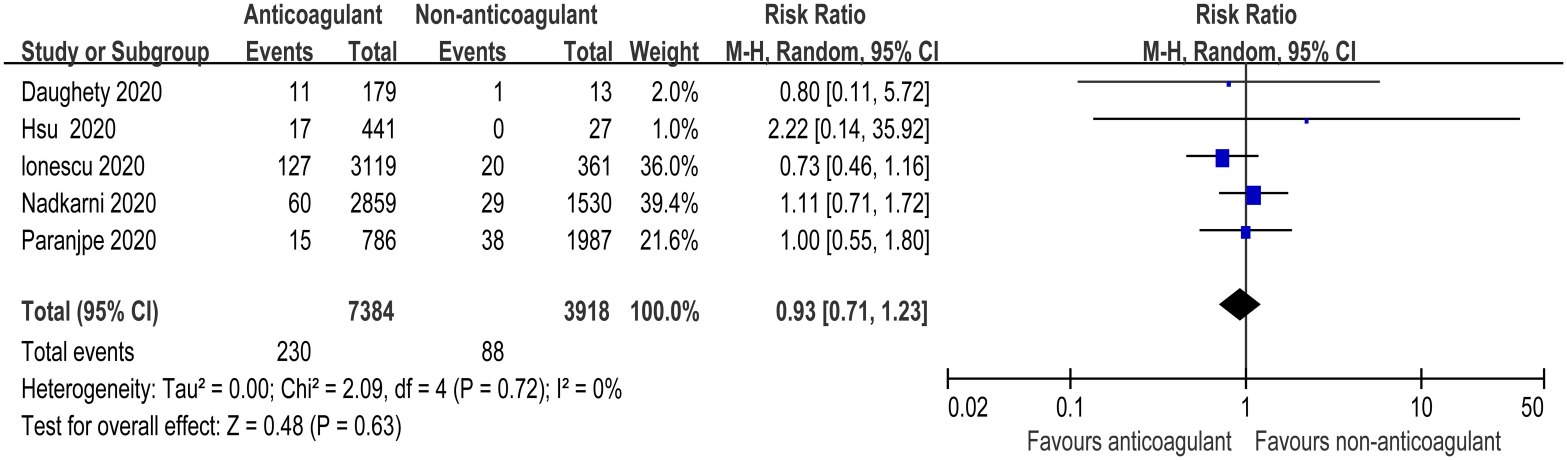
Forest plot of the effect of anticoagulants on bleeding events in hospitalized COVID-19 patients.

There were five studies that specified bleeding events during the anticoagulant therapy for COVID-19 patients (25, 27–29, 31). The meta-analysis with a total of 11,302 patients found that the pooled risk ratio of bleeding risk did not favor either of the two groups (RR = 0.93, 95% CI 0.71–1.23; *p* = 0.63, *I*^2^ = 0%, Figure 4). The quality of evidence for bleeding events was rated as moderate because plausible confounding is not considered to reduce or increase demonstrated effect (Table 3).

### Risk of Bias Assessment

**Figure 5:**
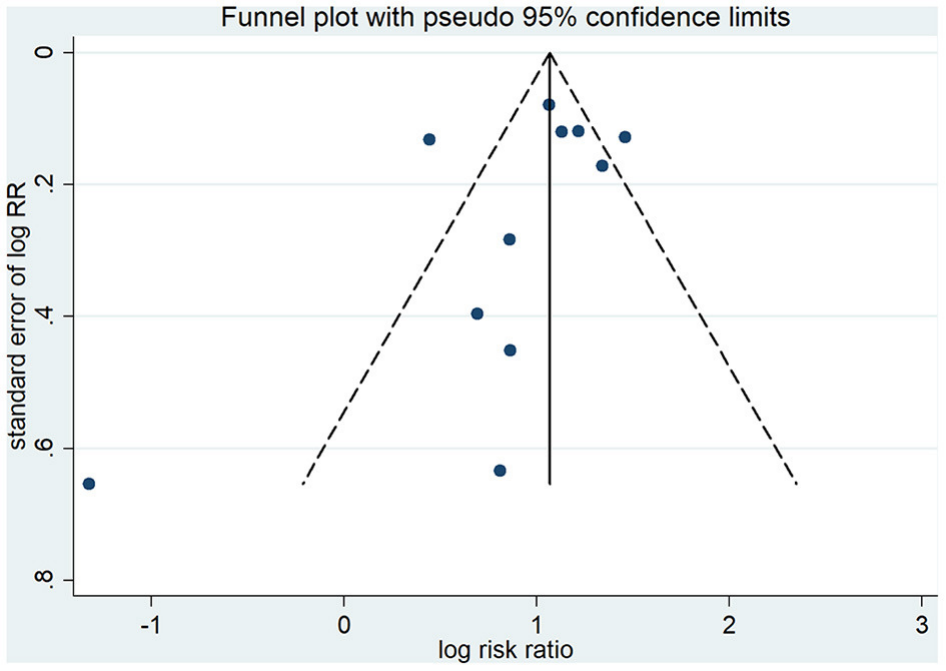
Funnel plot for the assessment risk of publication bias.

As shown in Figure 5, visual assessment of the funnel plot did show a little substantial asymmetry, but Begg's rank correlation test did not indicate the evidence of publication bias across studies of anticoagulant therapy in COVID-19 patients and mortality (*p* = 0.213).

### Sensitivity Analysis

In order to assess the stability of the results of the current meta-analysis, we first performed a one-study-removed sensitivity analysis for mortality. Statistically, similar results were obtained after omitting each of the studies (Table 4), indicating the stability of this meta-analysis. Given that the mortality of in-hospital COVID-19 patients differs significantly among countries due to multiple factors such as reporting bias, transparency, and definition of COVID-19 mortality, we further performed two exploratory sensitivity analyses to test the stability of the results in this meta-analysis. As shown in Table 5, the RR value of anticoagulation therapy was 0.75 (0.63, 0.89) when clinical studies performed in the USA were excluded. However, an insignificantly reduced in-hospital mortality risk was observed when trials conducted outside of the USA were excluded (RR = 0.67, 95% CI 0.43–1.02, *p* = 0.06, Table 6).

**Table 4:** Sensitivity analysis by omitting each study in random-effects model.

**Study omitted**	**RR (95% CI)**	***p***
Ayerbe et al. (26)	0.70 (0.51, 0.96)	0.03
Ionescu et al. (25)	0.78 (0.67, 0.91)	0.002
Daughety et al. (27)	0.69 (0.51, 0.92)	0.01
Reyes Gil et al. (30)	0.69 (0.51, 0.93)	0.01
Hsu et al. (28)	0.71 (0.52, 0.95)	0.02
Castelnuovo et al. (24)	0.69 (0.50, 0.96)	0.03
Nadkarni et al. (29)	0.68 (0.49, 0.93)	0.02
Paranjpe et al. (9)	0.67 (0.49, 0.92)	0.01
Rentsch et al. (10)	0.71 (0.52, 0.98)	0.04
Tang et al. (11)	0.67 (0.49, 0.91)	0.01
Zhang et al. (23)	0.70 (0.52, 0.96)	0.02

*RR, risk ratio; CI, confidence interval*.

**Table 5:** Sensitivity analysis by omitting studies performed outside of the USA in random-effects model.

**Studies from USA outside omitted**	**RR (95% CI)**	***p***
Ayerbe et al. (26)		
Di Castelnuovo et al. (24)	0.67 (0.43, 1.02)	0.06
Tang et al. (11)		
Zhang et al. (23)		

*RR, risk ratio; CI, confidence interval*.

**Table 6:** Sensitivity analysis by omitting studies from USA in random-effects model.

**Studies from the USA omitted**	**RR (95% CI)**	***p***
Daughety et al. (27)		
Reyes Gil et al. (30)		
Hsu et al. (28)		
Ionescu et al. (25)	0.75 (0.63, 0.89)	0.0009
Nadkarni et al. (29)		
Paranjpe et al. (9)		
Rentsch et al. (10)		

*RR, risk ratio; CI, confidence interval*.

## Discussion

This meta-analysis, including 11 observational studies with 20,748 in-hospital patients with COVID-19, demonstrated that anticoagulant administration significantly reduced the relative risk of mortality by 30%, with high-quality evidence as accessed by the GRADE framework and with confirmation using sensitivity analysis. The subgroup analyses showed anticoagulants could potentially reduce greater relative effect on mortality by 41% in trials with severe COVID-19 patients admitted to ICU. Bleeding events as measured by our criteria were relatively rare issues in anticoagulant therapy. These findings demonstrated that anticoagulant therapy could be used to reduce the risk of mortality in hospitalized patients with COVID-19, especially in critically ill patients, which are contrary to a previously published meta-analysis (32).

Results from previous researches investigating the role of anticoagulant administration among inpatients with COVID-19 have varied, which might be derived from different regimens of anticoagulation (e.g., drug type, dosage, and route), or different patient population (e.g., diverse disease severity), or disparate inclusion and exclusion criteria used in each study. For instance, early studies found no significant difference in mortality of COVID-19 patients with anticoagulation therapy (11, 12), and a previous meta-analysis confirmed this finding (32). But these studies were limited in sample size, and only 2,772 patients were included in the previous meta-analysis. In contrast, our review included data from 11 observational studies with more inpatients with COVID-19, which showed a significant association of anticoagulant use with 30% reduced hazard of in-hospital mortality in COVID-19 patients admitted to hospital. Limited evidence from only three studies with 2,672 participants also supported the use of heparin in critically ill COVID-19 patients admitted to ICU. Likewise, in 395 patients who required mechanical ventilation, Paranjpe et al. found that in-hospital mortality was 29.1% with a median survival of 21 days for those treated with anticoagulants as compared with 62.7% with a median survival of 9 days in patients who did not receive anticoagulants (9). Some researchers also argue that heparin, unlike other anticoagulants, may theoretically improve host survival in COVID-19 by exerting its potential anti-inflammatory (33–35) and direct antiviral effects (36–39).

Our sensitivity analyses further demonstrated that the overall effect of anticoagulant use was affected by the location of studies as evidenced by an insignificant beneficial effect found in the pooled analysis of the studies conducted in the USA. However, the results should be cautiously interpreted because this beneficial effect might be underestimated since we only extracted data of crude in-hospital mortality in each study for comparison during the data extraction process. For instance, the studies performed in the USA by Nadkarni et al. (*n* = 4,389) with significantly different baseline characteristics showed higher crude in-hospital mortality in anticoagulation group (28.6%) vs. non-anticoagulation group (25.6%), but a following analysis using inverse probability treatment weighted models for confounder adjustment inversely suggested that anticoagulation was associated with a lower adjusted risk of mortality vs. no anticoagulation (29).

Bleeding remains as a major safety concern when anticoagulant is administrated to COVID-19 patients with abnormal coagulation function. It is widely accepted that the potential benefits of systemic anticoagulation with heparin need to be weighed against the risk of bleeding and therefore should be individualized. In the current meta-analysis, bleeding events as measured by requirement for blood transfusions were not associated with the use of anticoagulants in COVID-19 patients, and major bleeding events were less common. Billett and colleagues did not find any anticoagulant regimen (prophylactic/therapeutic dose of apixaban, enoxaparin, and heparin) with a likelihood of transfusion greater than for patients on non-anticoagulation regimen either (40). Likewise, no signs were found of more subclinical bleeding in critically ill COVID-19 patients with higher doses of anticoagulation, perhaps because COVID-19 patients are hypercoagulable and might not bleed easily despite high-dose thromboprophylaxis (41).

Our study also has several limitations. First, the mean age of COVID-19 patients including our studies is almost over 60 years. Given that the mortality is significantly higher in the elderly hospitalized COVID-19 patients with more comorbid diseases, it will be better to include more studies with a broader age scope. Second, we extracted the raw data from the included studies even though the median survival days, sample sizes, and baseline characteristics were significant variants between different groups. Third, our meta-analysis was largely based on observational studies and might have been affected by allocation or selection bias.

## Conclusions

Among patients with COVID-19 admitted to hospital, the administration of anticoagulants was significantly associated with decreased in-hospital mortality without increasing the incidence of bleeding events. However, pragmatic trials and well-designed RCT studies with longer follow-up duration and larger sample size are warranted to further investigate the effect of anticoagulant therapy on the mortality of hospitalized COVID-19 patients in the real-world practice.

## Data Availability Statement

The original contributions generated for the study are included in the article/Supplementary Material, further inquiries can be directed to the corresponding author/s.

## Author Contributions

LJ and BS conceived the study and planned the content. LJ and YL drafted the manuscript and contributed equally to this work. BS reviewed and revised the manuscript. All authors contributed to the article and approved the submitted version.

## Conflict of Interest

The authors declare that the research was conducted in the absence of any commercial or financial relationships that could be construed as a potential conflict of interest.

## Publisher's Note

All claims expressed in this article are solely those of the authors and do not necessarily represent those of their affiliated organizations, or those of the publisher, the editors and the reviewers. Any product that may be evaluated in this article, or claim that may be made by its manufacturer, is not guaranteed or endorsed by the publisher.
