# Supplementary material for: Effect of Anticoagulant Administration on the Mortality of Hospitalized Patients With COVID-19: An Updated Systematic Review and Meta-Analysis
Source: Front Med (Lausanne). 2021 Aug 4;8:698935. doi: 10.3389/fmed.2021.698935 (PMC8371681; doi:10.3389/fmed.2021.698935)
Supplement: Supplementary file 1 [file Table_1.DOCX]

| **Section/topic** | **#** | **Checklist item** | **Reported on page #** |  |  |
| --- | --- | --- | --- | --- | --- |
| **TITLE** | | |  |  |  |
| Title | 1 | Identify the report as a systematic review. | 1 |  |  |
| **ABSTRACT** | | |  |  |  |
| Abstract | 2 | Provide a structured summary including, as applicable: background; objectives; data sources; study eligibility criteria, participants, and interventions; study appraisal and synthesis methods; results; limitations; conclusions and implications of key findings; systematic review registration number. | 2 |  |  |
| **INTRODUCTION** | | |  |  |  |
| Rationale | 3 | Describe the rationale for the review in the context of what is already known. | 3 |  |  |
| Objectives | 4 | Provide an explicit statement of questions being addressed with reference to participants, interventions, comparisons, outcomes, and study design (PICOS). | 3 |  |  |
| **METHODS** | | |  |  |  |
| Protocol and registration | 5 | Indicate if a review protocol exists, if and where it can be accessed (e.g., Web address), and, if available, provide registration information including registration number. | 4 |  |  |
| Eligibility criteria | 6 | Specify the inclusion and exclusion criteria for the review and how studies were grouped for the syntheses. | 5 |  |  |
| Information sources | 7 | Specify study characteristics (e.g., PICOS, length of follow-up) and report characteristics (e.g., years considered, language, publication status) used as criteria for eligibility, giving | 4 |  |  |
| Search strategy | 8 | Present full electronic search strategy for at least one database, including any limits used, such that it could be repeated. | Supplementary Table 2 |  |  |
| Selection process | 9 | State the process for selecting studies (i.e., screening, eligibility, included in systematic review, and, if applicable, included in the meta-analysis). | 4 |  |  |
| Data collection process | 10 | Describe method of data extraction from reports (e.g., piloted forms, independently, in duplicate) and any processes for obtaining and confirming data from investigators. | 5 |  |  |
| Data items | 11 | List and define all variables for which data were sought (e.g., PICOS, funding sources) and any assumptions and simplifications made. | 5 |  |  |
| Study risk of bias assessment | 12 | State the principal summary measures (e.g., risk ratio, difference in means). | 6 |  |  |
| Effect measures | 13 | Specify for each outcome the effect measure(s) (e.g. risk ratio, mean difference) used in the synthesis or presentation of results. | 6 |  |  |
| Additional analyses | 14 | Describe any sensitivity analyses conducted to assess robustness of the synthesized results. | 6 |  |  |
| Reporting bias assessment | 15 | Describe any methods used to assess risk of bias due to missing results in a synthesis (arising from reporting biases). | 6 |  |  |
| Certainty assessment | 16 | Describe any methods used to assess certainty (or confidence) in the body of evidence for an outcome. | 6 |  |  |
| **RESULTS** | | |  |  |  |
| Study selection | 17 | Give numbers of studies screened, assessed for eligibility, and included in the review, with reasons for exclusions at each stage, ideally with a flow diagram. | 7 |  |  |
| Study characteristics | 18 | For each study, present characteristics for which data were extracted (e.g., study size, PICOS, follow-up period) and provide the citations. | 7 |  |  |
| Risk of bias in studies | 19 | Present data on risk of bias of each study and, if available, any outcome level assessment | 8 |  |  |
| Results of individual studies | 20 | For all outcomes considered (benefits or harms), present, for each study: (a) simple summary data for each intervention group (b) effect estimates and confidence intervals, ideally with a forest plot. | 8-9 |  |  |
| Results of syntheses | 21 | Present results of each meta-analysis done, including confidence intervals and measures of consistency. | 8-9 |  |  |
| Reporting biases | 22 | Present results of any assessment of risk of bias across studies. | 9 |  |  |
| Certainty of evidence | 23 | Give results of additional analyses, if done (e.g., sensitivity or subgroup analyses, meta-regression [see Item 16]). | 9-10 |  |  |
| **DISCUSSION** | | |  |  |  |
| Discussion | 24 | Summarize the main findings including the strength of evidence for each main outcome; consider their relevance to key groups (e.g., healthcare providers, users, and policy makers). | 10 |  |  |
|  | 25 | Discuss limitations at study and outcome level (e.g., risk of bias), and at review-level (e.g., incomplete retrieval of identified research, reporting bias). | 11-12 |  |  |
|  | 26 | Provide a general interpretation of the results in the context of other evidence, and implications for future research. | 12 |  |  |
|  | 27 | Discuss implications of the results for practice, policy, and future research. | 13 |  |  |
| **OTHER INFORMATION** | | |  |  |  |
| Funding | 28 | Describe sources of funding for the systematic review and other support (e.g., supply of data); role of funders for the systematic review. | 13 |  |  |

*From:* Moher D, Liberati A, Tetzlaff J, Altman DG. The PRISMA Group (2009). Preferred Reporting Items for Systematic Reviews and Meta-Analyses: The PRISMA Statement. PLoS Med 6(7): e1000097. doi:10.1371/journal.pmed1000097.
