# Supplementary material for: Effect of Anticoagulant Administration on the Mortality of Hospitalized Patients With COVID-19: An Updated Systematic Review and Meta-Analysis
Source: Front Med (Lausanne). 2021 Aug 4;8:698935. doi: 10.3389/fmed.2021.698935 (PMC8371681; doi:10.3389/fmed.2021.698935)
Supplement: Supplementary file 3 [file Table_3.DOCX]

| **Supplementary table 3:** Anticoagulant protocols of studies fulfilling the inclusion criteria | | |
| --- | --- | --- |
| **Author, year** | **Observation group** | **Control group** |
| Ayerbe et al, 2020 ^[21]^ | AC: heparin, dose unknown. | NAC: non-anticoagulation. |
| Castelnuovo et al, 2021 ^[19]^ | AC: TAC+PAC  TAC: LMWH at dose of 6000 IU/day if body weight＜100 kg and GFR＜30mL/min.  PAC: LMWH at dose of 6000 IU/day if body weight >100 kg and GFR＞30mL/min. | NAC: non-anticoagulation. |
| Daughety et al, 2020 ^[22]^ | AC: TAC+PAC  TAC: 1) enoxaparin at dose of 1mg/kg twice per day; 2) intravenous UFH titrated to anti-factor Xa levels 0.5–0.7 U/mL in patients with renal failure.  PAC: 1) enoxaparin at dose of 40 mg once per day (<100kg) or 60 mg once per day (>100kg); 2) UFH at dose of 5000 U thrice per day in patients with renal failure. | NAC: non-anticoagulation. |
| Gil et al, 2020 ^[25]^ | AC: TAC+PAC  TAC: anticoagulant type and dose unknown.  PAC: anticoagulant type and dose unknown. | NAC: non-anticoagulation. |
| Hsu et al, 2020 ^[23]^ | AC: TAC+PAC.  TAC: 1) intravenous heparin; 2) LMWH at dose of 1 mg/kg twice per day; 3) dose-adjusted warfarin with a target international normalized ratio of 2.0 to 3.0; 4) apixaban at dose of 5 mg twice per day; 5) rivaroxaban at dose of 20 mg once per day.  PAC: 1) LMWH at dose of 40 mg once per day; 2) subcutaneous UFH at dose of 5000 U thrice per day; 3) apixaban at dose of 2.5 mg twice per day. | NAC: non-anticoagulation. |
| Ionescu et al, 2020 ^[20]^ | AC: TAC+PAC.  TAC: a minimum 3-day course of either: 1) intravenous UFH with at least one documented activated partial thromboplastin time ≥ 45 s; 2) subcutaneous enoxaparin at doses of 1 mg/kg twice per day or 1.5 mg/kg once per day; 3) intravenous argatroban infusion; 4) subcutaneous fondaparinux at doses of 5-10 mg once per day.  PAC: 1) subcutaneous UFH at doses of 5000 units twice or three times per day; 2) subcutaneous enoxaparin injection at doses of 30-40 mg once per day; 3) subcutaneous fondaparinux at a dose of 2.5 mg once per day; 4) Therapeutic-dose anticoagulation for less than 3 days. | NAC: non-anticoagulation. |
| Nadkarni et al, 2020 ^[24]^ | AC: TAC+PAC.  TAC: enoxaparin at dose of 1mg/kg twice per day.  PAC: enoxaparin at dose of 30-40 mg once per day. | NAC: non-anticoagulation. |
| Paranjpe et al, 2020 ^[26]^ | AC: anticoagulant type and dose unknown. | NAC: non-anticoagulation. |
| Rentsch et al, 2021 ^[7]^ | AC: AC+PAC.  TAC: 1) intravenous heparin variable use partial thromboplastin time; 2) subcutaneous enoxaparin at dose of > 40 mg 40 mg once per day; 3) subcutaneous fondaparinux at doses of 5, 7.5, 10 mg once per day; 4) subcutaneous dalteparin at dose of ≥5500 IU twice per day;5) oral apixaban at dose of 5 mg twice per day; 6) oral rivaroxaban at dose of ≥ 15 mg once per day; 7) oral dabigatran at dose of 150 mg twice per day; 8) oral edoxaban at dose of 60 mg once per day; 9) oral warfarin variable use international normalized ratio.  PAC: 1) subcutaneous heparin at dose of 5000 units twice or thrice per day; 2) subcutaneous enoxaparin at dose of 40 mg once per day or 30 mg twice per day; 3) subcutaneous fondaparinux at dose of 2.5 mg once per day; 4) subcutaneous dalteparin at dose of 2500-5000 IU once per day; 5) oral apixaban at dose of 2.5 mg twice per day; 6) oral rivaroxaban at dose of 10 mg once per day or 2.5 mg twice per day for arterial disease; 7) oral dabigatran at dose of 220 mg once per day | NAC: non-anticoagulation |
| Tang et al, 2020 ^[8]^ | AC: 1) enoxaparin at dose of 40-60 mg once per day; 2) UFH at dose of 10 000-15 000 IU once per day | NAC: non-anticoagulation |
| Zhang et al, 2020 ^[18]^ | AC: LMWH, dose unknown | NAC: non-anticoagulation |

**Abbreviation:** AC, anticoagulation; NAC, non- anticoagulation；PAC, prophylactic-dose anticoagulation; TAC, therapeutic-dose anticoagulation; GFR，glomerular filtration rate; LMWH, low-molecule-weight heparin; UFH, unfractioned heparin
